# Catalytic Effect of Photoluminescent Zinc Oxide Nanoparticles Formed in the Presence of Quaternary Ammonium Salts

**DOI:** 10.3390/ma12132066

**Published:** 2019-06-27

**Authors:** Aurel Tăbăcaru, Rodica Mihaela Dinică, Mihaela Cudălbeanu, Cristina Mihaela Nicolescu, Marius Bumbac

**Affiliations:** 1 Faculty of Sciences and Environment, Department of Chemistry, Physics and Environment, “Dunarea de Jos” University of Galati, 111 Domneasca Street, 800201 Galati, Romania; 2 Institute of Multidisciplinary Research for Science and Technology, Valahia University of Targoviste, 13 Aleea Sinaia, 130004 Targoviste, Romania; 3 Faculty of Sciences and Arts, Department of Sciences and Advanced Technologies, Valahia University of Targoviste, 13 Aleea Sinaia, 130004 Targoviste, Romania

**Keywords:** zinc oxide nanoparticles, surface modification, quaternary ammonium salts, assisted organic synthesis, fluorescence

## Abstract

The comparative effect of two quaternary ammonium salts from 1,2-bis(4-pyridyl)ethane (PyQAs), namely N,N′-diphenacyl-1,2-bis(4-pyridinium)ethane dibromide (PyQAs1) and N,N′-di(*p*-methoxyphenacyl)-1,2-bis(4-pyridinium)ethane dibromide (PyQAs2), upon the size and photoluminescence of zinc oxide nanoparticles (ZnO NPs) was investigated. The formation of ZnO NPs took place in the presence of variable amounts of the two PyQAs species (1, 2.5, and 5%), according to the chemical precipitation of zinc(II) acetate with potassium hydroxide in ethanol under reflux. The obtained ZnO NPs were structurally characterized by means of X-ray powder diffraction, infrared, and Raman spectroscopy. The fluorescence of all supernatant solutions, observed under ultraviolet light, determined us to make an investigation of the solutions by means of liquid chromatography coupled with electrospray ionization mass spectrometry (LC-MS-ESI) in order to elucidate the identity of the newly formed fluorescent species. Such an occurrence thus allowed the invocation of the catalytic effect of zinc(II) ions towards the organic transformation of both nonfluorescent PyQAs surfactants into new fluorescent organic species.

## 1. Introduction

Zinc oxide (ZnO) is a semiconductor material of group II–VI, with several special properties, such as wide band gap energy (3.37 eV) and large exciton-binding energy (60 meV) at room temperature, along with high electron mobility, elevated transparency, and photoluminescence [1]. These properties allowed ZnO to receive a central place as multifunctional material among different emerging technological applications in, e.g., optics, magnetism, and electronics [2]. ZnO has also proved a great versatility as it can be obtained through many possible synthetic methodologies [3,4], assuring multiple morphologies and sizes that go from micro- to nanoscale [5,6]. Due to the large variety of physico-chemical properties induced by the multiple morphologies and sizes thus obtained, in the last decade numerous potential and practical applications have been found for ZnO spanning from optoelectronics [7] to solar cells [8], thin-film transistors [9], catalysis [10], sensing [11], biomedicine [12], and antibacterial agents [13].

The successful integration into some of these practical applications requires the utilization of ZnO in the form of very small particles which, in the absence of surface modifiers, they would tend to agglomerate due to the so-called Ostwald ripening [14]. On the other hand, the poor chemical stability and the intrinsic defects of ZnO nanomaterials are important factors that must be taken into consideration, in order to attain sustainable performances for the above-mentioned applications. That is why the control upon the surface properties of ZnO nanomaterials, through different surface functionalization approaches, has become crucial and beneficial in the same time, not only for the enhancement and sustainability of their performances, but also for the generation of new properties and functions [15,16,17].

Surface modification of ZnO nanomaterials with carefully chosen organic surfactants also brings the advantage of reaching highly stable colloidal solutions in which the growth and surface defects of ZnO particles can be successfully suppressed [18,19,20]. Consequently, a size reduction effect depending on the concentration of surfactants can be reached even at the level of quantum dots, which in turn can finely tune both morpho-structural and electronic features [21,22]. For instance, surface modification of ZnO NPs through functionalization with increasing concentrations of alkoxysilane molecules led to significant size-decreased particles, for which a fine tuning of the photoemission wavelengths was attained [23,24,25,26]. Other organic surfactants, though limited in number and diversity, have also been successfully introduced into the surface modification practice of ZnO NPs, such as polymers [27], alkylamines [28], acrylates [29], thiols [30], or species bearing long alkyl chains [31]. 

**Figure 1:**
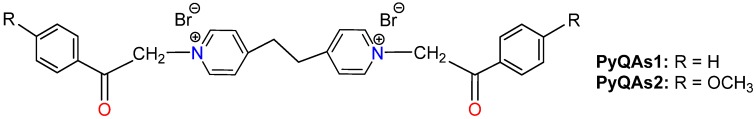
Structure of the two diquaternary salts of bis(pyridinium) (PyQAs) used in the assisted formation of ZnO NPs.

In the context of surface modification of ZnO NPs, we here focus on the diversification of such a practice by employing, for the first time, a particular category of cationic surfactants from the class of quaternary ammonium salts bearing the 1,2-bis(4-pyridyl)ethane core (PyQAs), namely N,N′-diphenacyl-1,2-bis(4-pyridinium)ethane dibromide (PyQAs1) and N,N′-di(*p*-methoxyphenacyl)-1,2-bis(4-pyridinium)ethane dibromide (PyQAs2) (Figure 1). The choice of utilizing such PyQAs surfactants was initiated by the presence of carbonyl and methoxy functional groups that could be involved in chemical bonding with the zinc(II) ions and subsequent surface modification during the formation of ZnO NPs. 

The present paper is therefore aimed at investigating the comparative effect of the two above-mentioned PyQAs surfactants, used in variable amounts, upon the size and photoluminescence of two new series of ZnO NPs, which were obtained by the chemical precipitation method in alcoholic ambient. It is worth mentioning that the chemical synthesis approach has a generally inherent drawback of contributing unintentional impurities to the synthesized products. The liquid containing nanomaterials produced by using chemical routes may always contain other ions and reaction by-products that are difficult to separate from the liquid, and therefore, the products are not contamination-free. That is why green synthesis alternatives to chemical routes have started to successfully deal with such issues [32]. 

While X-ray powder diffraction was used to confirm the formation of ZnO phase, infrared and Raman spectroscopy were used to verify the presence and eventual chemical attachment of PyQAs surfactants on the ZnO surface. Surprisingly, the fluorescence of all supernatant solutions, observed under UV light, determined us to make a preliminary investigation of the solutions by means of liquid chromatography coupled with electrospray ionization mass spectrometry (LC-MS-ESI) in order to elucidate the identity of the newly formed fluorescent species. Such an occurrence thus allowed the invocation of the catalytic effect of zinc(II) ions towards the organic transformation of both nonfluorescent PyQAs species into new fluorescent organic species.

## 2. Materials and Methods

### 2.1. General

- Zinc(II) acetate dihydrate (>98%), potassium hydroxide and ethanol (96%) were purchased from Sigma-Aldrich and used as received.
- N,N′-diphenacyl-1,2-bis(4-pyridinium)ethane dibromide (PyQAs1) and N,N′-di(*p*-methoxyphenacyl)-1,2-bis(4-pyridinium)ethane dibromide (PyQAs2) were synthesized according to the method previously reported in the literature [33].
- X-ray diffraction (XRD) technique was used to record X-ray patterns of dried ZnO samples
- Data acquisition was performed with a Ultima IV diffractometer (Rigaku, Tokyo, Japan), using a monochromatized Cu Kα radiation (λ = 1.54056 Å), from a fixed anode X-ray tube operated at 40 kV and 20 mA. Diffractograms were recorded for the 2-theta range of 5–120 degrees, in a parallel beam geometry with continuous scan mode, at 5 degrees/minute speed and 0.02 degrees step width. Crystal structure analysis was performed using the functionalities of the software PDXL v. 2.2. and ICDD data base PDF4+ v. 2015.
- Fourier Transform Infrared Spectroscopy (FTIR) was used to identify functional groups of organic compounds existing in studied samples, IR spectra were recorded on a Vertex 80 infrared spectrometer (Bruker, Ettlingen, Germany) in the wavenumbers range of 4000 cm^−1^ to 400 cm^−1^, with 32 scans per sample. Three KBr pellets were prepared to be scanned with aliquots weighted from each sample type. The same mixing ratio used to prepare the pellets was of 2.5% (w/w) sample to potassium bromide.
- Raman spectra were recorded at 1064 nm on Xantus 2 spectrometer (Rigaku, Tokyo, Japan), in the wavelength range of 200–2000 cm^−1^, with a spectral resolution of 7–10 cm^−1^.The total exposure time of a Raman spectrum was 1000 milliseconds, and the laser power used was 300 mW. Instrument calibration was made with pure acrylonitrile before each set of measurements. No specific sample preparation was needed, three samples of each type were scanned, and the average Raman spectra were calculated and then commented relative to the reference ZnO synthesized sample.
- Ultraviolet–visible (UV–vis) absorption spectra were recorded on Evolution 260 BIO spectrophotometer (Thermo Fischer Scientific, Waltham, MA, USA) for ethanolic saturated solutions of zinc oxide nanoparticles, placed in 1 cm quartz cuvettes. The absorption spectra were recorded in the range of 200–1100 nm, at 1 nm intervals, at the same integration time and using ethanol solvent as blank, then relevant plots of 350–450 nm were included and commented.
- Solid state photoluminescence spectra were recorded with a Microplate reader with fluorescence spectrometer Infinite 200 PRO NanoQuant (Tecan, Männedorf, Switzerland), in the 400–800 nm range, upon excitation at 365 nm.
- The LC-MS-ESI analysis was carried out using a Bruker Esquire 3000 Plus Ion Trap Mass Spectrometer (Bruker Daltonics, Hamburg, Germany). In the chromatographic analysis, the C18 (150 × 4.6 mm, particle size of 5 μm) column was used with an injection volume of 25 µL. The solvents used were (A) formic acid (1%) and (B) methanol. For the mass spectra, the ionization source temperature was 500 °C and the mass spectra were recorded in the negative and positive ion mode between 0 and 800 m/z.

### 2.2. Synthesis of ZnO NPs: General Procedure

In 20 mL of ethanol were introduced variable amounts of PyQAs, in order to reach the Zn/PyQAs molar ratios of 0, 1, 2.5, and 5%, in a 100 mL round bottomed flask. The obtained solutions were heated until 60 °C for 10 minutes, and 2.45 g of zinc acetate dihydrate was added into the reaction vessel with stirring. A basic solution of 1.25 g of potassium hydroxide, separately dissolved in 10 mL of ethanol, was added dropwise. The cream suspensions so obtained were stirred for 3 h under reflux, and after this time, they were centrifuged (9000 rpm, 10 °C, 5 min), washed with three portions of ethanol (10 mL), and finally dried in air at 100 °C for several hours. The obtained powder samples are hereafter denoted with ZnO-PyQAs1-x% and ZnO-PyQAs2-x%, where x = 1, 2.5, and 5, is the Zn/PyQAs molar ratio used in the syntheses, whereas the reference sample, prepared in the same conditions and in the absence of PyQAs, is simply denoted with ZnO.

## 3. Results and Discussion

### 3.1. Synthesis

**Figure 2:**
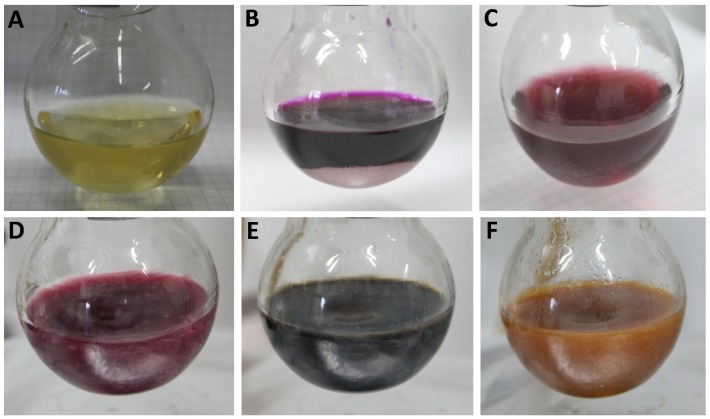
Aspect of the solutions in the first moments of syntheses: (**A**) ethanol solution containing PyQAs; (**B**) ethanol solution with PyQAs and zinc(II) acetate; (**C**) color change of solution (B) after heating and stirring for 10 minutes; (**D**–**F**) color change upon the addition of potassium hydroxide.

**Scheme 1:**
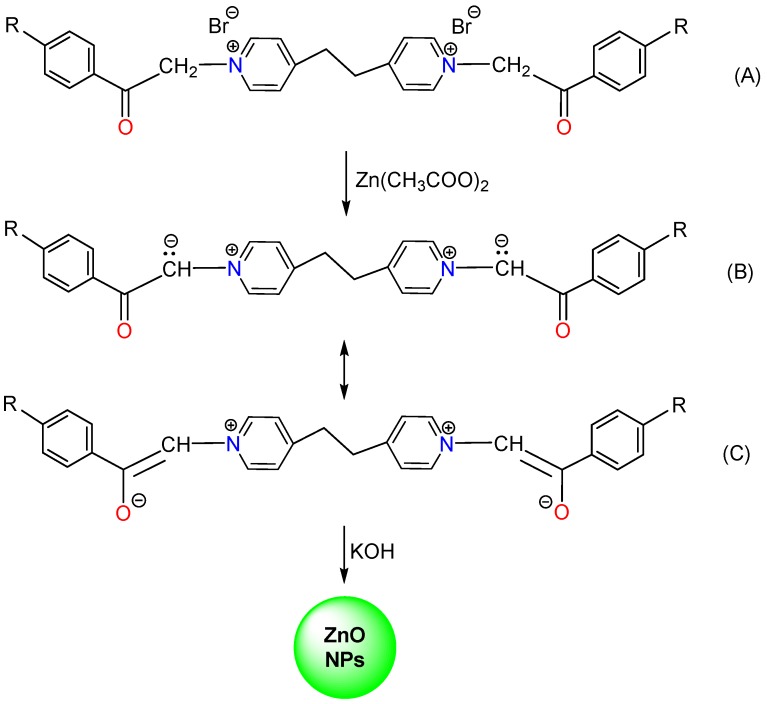
Reaction steps towards the formation of ZnO NPs assisted by PyQAs species.

The interaction of the two PyQAs cationic surfactants, used in variable amounts, with zinc(II) acetate, in ethanol under reflux, afforded two new series of ZnO NPs (Scheme 1), which were obtained by the chemical precipitation with potassium hydroxide. It is interesting to note that in the first moments of synthesis, all the solutions had different aspects (Figure 2), which led us to make some pertinent observations and interpretations related to the in-situ transformation of both PyQAs species within the formation process of ZnO NPs. 

Thus, after the dissolution of PyQAs in ethanol, heated to 60 °C, the color of the solution suddenly changed from yellowish (Figure 2A) to dark purple upon the addition of zinc(II) acetate dihydrate (Figure 2B). Such a color change was associated to the transformation of PyQAs species into their pyridinium ylide intermediates, promoted by the basic conditions provided, in the present case, by the acetate groups [34]. Further heating and stirring, lasting 10 min, led to the complete dissolution of zinc(II) acetate, concomitantly with the color change from dark purple to dark red (Figure 2C), possibly due to the complexation of the Zn^2+^ ions by the ylide intermediates through the enolate oxygen atoms. Slow addition of an ethanol solution containing potassium hydroxide led to a new progressive color change from dark red, through a very dark blue one, to light brown (Figure 2D–F). This color change was concomitantly accompanied by the progressive formation of a white precipitate, moment in which the strong basic ambient ensured by potassium hydroxide enables the hydrolysis of zinc(II)-enolate oxygen bonds towards the formation of zinc(II) hydroxide. The latter, in turn, undergoes polycondensation through water elimination between the hydroxyl groups to finally generate the particles identified as ZnO. After three hours of continuous stirring and heating, the white precipitates were isolated by centrifugation, dried in vacuo at 100 °C, and subjected to structural characterization by means of X-ray diffraction, infrared, and Raman spectroscopy. 

### 3.2. Structural Characterization

**Figure 3:**
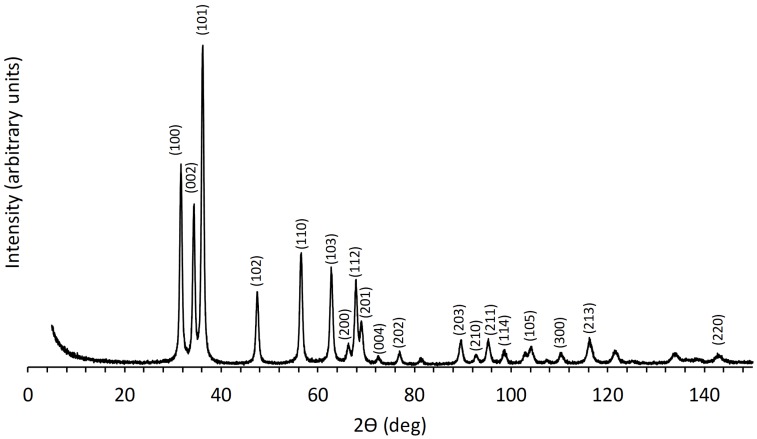
Measured diffractograms of samples ZnO, ZnO-PyQAs1-1%, ZnO-PyQAs1-2.5%, and ZnO-PyQAs1-5% (overlapping).

**Figure 4:**
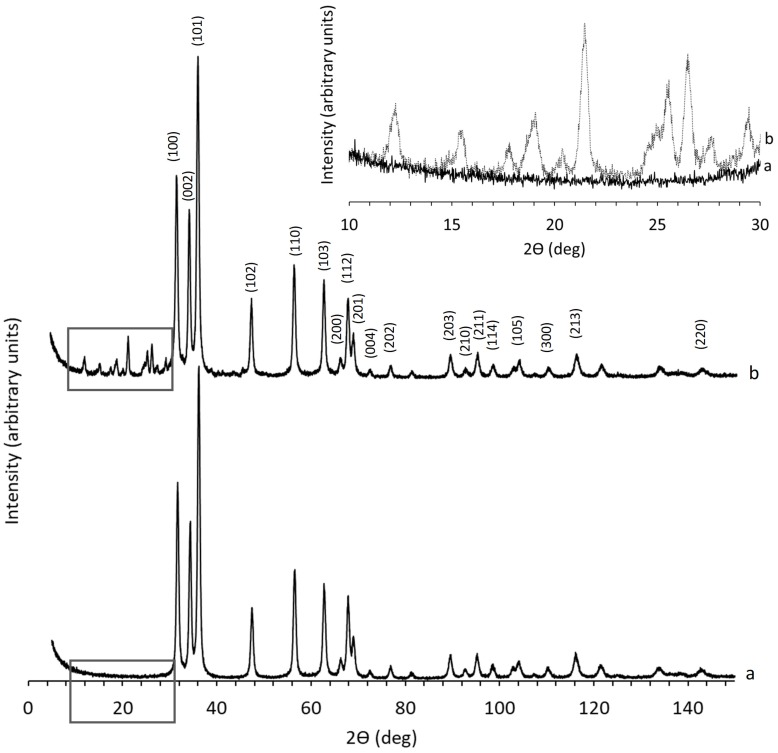
X-ray patterns recorded for samples ZnO, ZnO-PyQAs2-1% and ZnO-PyQAs2-2.5%—**a** (overlapped), and ZnO-PyQAs2-5%—**b**. Inset shows differences between a and b patterns in squared lined areas.

X-ray diffraction technique was used in the present study to confirm the presence of ZnO particles in the synthesized samples, to estimate their average crystallite size, and to compare their crystallinity. Figure 3 and Figure 4 show the XRD patterns of the ZnO samples prepared as described in the Experimental section. It may be observed that, for all studied samples, the recorded peaks are rather sharp, which may be attributed to their notable crystalline character. For the samples synthetized through the recipe utilizing the organic agent PyQAs1, the measured diffractograms overlapped the pattern recorded for the reference sample ZnO. On the other hand, the samples prepared using the organic agent PyQAs2 showed similar XRD patterns as the reference sample ZnO. Slight differences were observed for sample ZnO-PyQAa2-5%, its XRD pattern showing additional peaks in the 2-theta range of 10 to 30 degrees, as highlighted in the inset of Figure 4.

The crystal structure analysis performed in the present study confirmed the presence of crystalline ZnO phase in all synthesized samples. In Figure 3 and Figure 4, the XRD patterns illustrated for all studied samples showed clear distinct peaks that could be indexed as of hexagonal zincite structure found in the standard reference data (ICDD-pdf card no. 01-076-0704). Thus, the diffraction peaks observed at the 2-theta angles (in degrees) of ≈ 31.7, 34.3, 36.2, 47.4, 56.5, 62.7, 66.3, 67.8, 69.0, 72.4, 76.9, 89.6, 92.7, 95.2, 98.4, 104.1, 110.3, and 116.1 were attributed to the diffraction lines of (100), (002), (101), (102), (110), (103), (200), (112), (201), (004), (202), (203), (210), (211), (114), (105), (300), and (213), respectively. All the studied samples showed these peaks, all having rather low values of full width at half maximum (FWHM). Sample ZnO-PyQAs2-5% showed additional peaks at the 2-theta angles lower than 30 degrees, which were attributed to unknown phases.

The average crystallite size of the synthesized ZnO NPs was estimated by applying Scherrer’s equation

(1) $$D=\frac{k\cdot \lambda }{\beta \cdot cos\theta }$$

where *D* represents the crystallite size in nanometers, *θ* is the diffraction angle, *λ* is the wavelength of Cu-Kα radiation (0.15406 nm), *β* is the full width at half maximum (FWHM) for the peak considered, and *k* is the Sherrer’s constant whose value for spherical particles is 0.89 [35].

Table 1 and Table 2 present the comparative values of both the average crystallite size and FWHM, corresponding to the highest peak with Miller indices (101). A first clear effect of the two surfactants is thus shown by the attained sizes of formed wurtzitic ZnO NPs, as was revealed by the XRD investigations. As such, in the series of ZnO NPs obtained in the presence of PyQAs1 surfactant (methoxy-free), a significant variation of the average crystallite size values, from 14.666 nm in unmodified ZnO to 22.966 nm in ZnO modified with PyQAs1-2.5%, was observed. Instead, in the series of ZnO NPs modified obtained in the presence of PyQAs2 surfactant (methoxy-containing), the effect of the latter looks almost insignificant, as the variation of the average crystallite size values, comprised in the narrow range 13.998–15.666 nm, was observed. These results are therefore giving rise to make a first assessment of the comparative effect of the two PyQAs surfactants upon the crystallite size variation of formed ZnO NPs.

By comparing the crystallite size and FWHM values, it is noticeable that the PyQAs2 surfactant values do not differ when surfactant concentration is modified. When PyQAs1 surfactant was used for the synthesis of ZnO NPs, the values were different when the organic compound concentration exceeded 2.5%. Crystallite size may be different due to inhomogeneous strain. Crystallite size may differ if crystal microstructure changes due to phenomena like dislocation generation, bending, point defect, diffusion [36].

**Figure 5:**
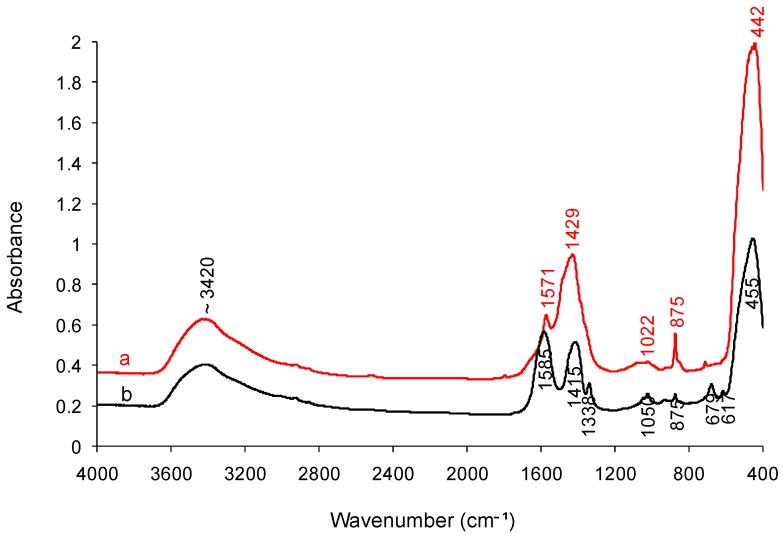
FTIR spectra of ZnO NPs obtained without PyQAs1 (**a**) and with 5% PyQAs1 (**b**).

**Figure 6:**
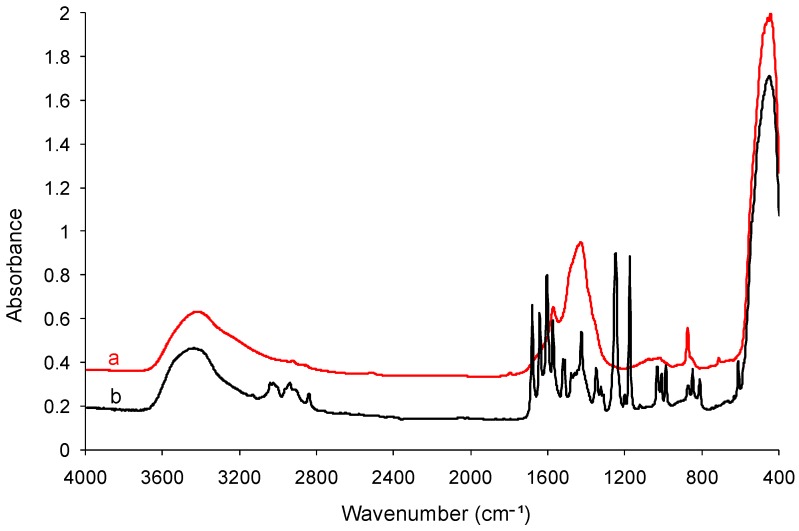
FTIR spectra of ZnO NPs obtained without PyQAs2 (**a**) and with 5% PyQAs2 (**b**).

Fourier transform infrared spectroscopy (FTIR) and its complementary technique, Raman spectroscopy, were used to verify the presence of PyQAs cationic surfactants employed in the surface modification of synthesized ZnO NPs. Figure 5 and Figure 6 gather the FTIR spectra of unmodified ZnO NPs and ZnO NPs modified with 5% PyQAs species, while Table 3 gathers all the corresponding absorbtion bands, along with their specific assignments.

In both mentioned figures, the absorption peaks observed at 442 cm^−1^ for the unmodified ZnO NPs and 455 cm^−1^ for ZnO NPs modified with PyQAs surfactants are specific to the vibration of zinc–oxygen bonds [39]. The absorption peaks around the values 875, 1420, and 1570 cm^−1^ could be assigned to the acetate group that remained attached onto the surface of ZnO NPs even after insistent washings with ethanol, and the absorption band at 3420 cm^−1^ was assigned to the O–H stretching vibrations from the physisorbed water molecules [40]. Apparently, no specific bands of the PyQAs species were observed in the case of all ZnO samples, but in the one modified with 5% PyQAs2.

**Figure 7:**
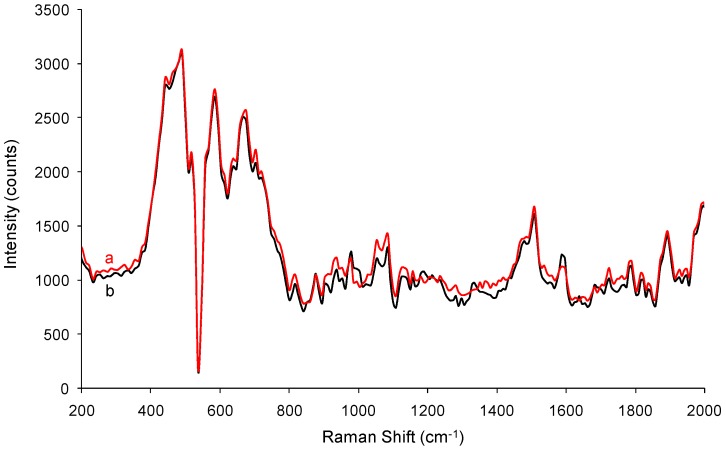
Raman spectra of ZnO NPs obtained without PyQAs1 (**a**) and with 5% PyQAs1 (**b**).

**Figure 8:**
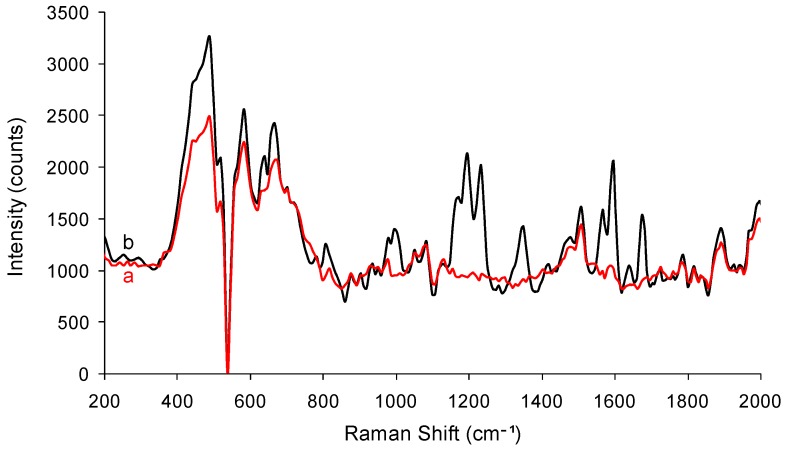
Raman spectra of ZnO NPs obtained without PyQAs2 (**a**) and with 5% PyQAs2 (**b**).

Figure 7 and Figure 8 gather the Raman spectra of unmodified ZnO NPs and ZnO NPs modified with 5% PyQAs species, while Table 4 comprises the Raman peaks and the corresponding assignments. In the Raman spectra of ZnO NPs modified with PyQAs1 (Figure 7) slight differences in the 1100–1500 cm^−1^ range could be observed, a range of bands characteristic to ring vibrations, to C‒C stretching vibrations for sp^3^ carbon, and also to ‒CH_2_‒twisting vibrations. Also, slight differences between the positions of Raman scattering bands of the two sample types may be observed in the range of 1700–1800 cm^−1^, a phenomenon that may be explained by the presence of carbonyl groups from the structure of PQAs1. Thus, the presence of such bands suggests that the formed ZnO NPs are accompanied by negligible traces of PyQAs1, not evidenced in the FTIR spectra, which are either in a coordinated form or adsorbed on the nanoparticles’ surface.

In the Raman spectra of ZnO NPs modified with PyQAs2 (Figure 8) significant differences between the patterns of unmodified ZnO NPs and ZnO NPs modified with 5% PyQAs2 could be observed. Peaks specific to ZnO NPs (in 400–600cm^−1^ domain) do not change their position no matter what the synthesis route was chosen (Table 4). In addition, it is noticed that the peak of ~810 cm^−1^, attributable to CH_2_ rocking vibrations, changes to 876 cm^−1^ when PyQAs2 is used, and in the ZnO NPs spectrum a peak at 1435 cm^−1^ appears, thus indicating the presence of the methoxy group in the structure of ZnO NPs formed when PyQAs2 surfactant was used. Other notable bands may be observed in the Raman spectrum of sample ZnO-PyQAs2-5%: stretching vibrations for C–O, ‒CH_2_‒ twisting vibrations, aromatic ring vibrations, C=O stretching and deformation vibrations [41,42]. By comparing all Raman spectra, one may observe that more significant differences between the two experimental setups—without versus with surfactant addition—were recorded for the case when PyQAs2 was used in the synthesis process of ZnO NPs. Thus, a second clear effect of the surfactants is also shown by FTIR and Raman spectroscopic investigations, which indicated stronger bonding between the ZnO surface and the methoxy-containing PyQAs2 surfactant. The presence of methoxy activating group in the *para* position of the aromatic rings may give rise to provide additional bonding sites for the Zn^2+^ ions, thus providing a higher coverage degree of the ZnO surface [43].

As a final remark, the absence of all specific absorption bands related to the PyQAs structures, from both infrared and Raman spectra of the formed ZnO NPs modified with 1 and 2.5% PyQAs surfactants, indicated the fact that these cationic surfactants were not attached onto the surface of ZnO NPs. 

### 3.3. Optical Properties

**Figure 9:**
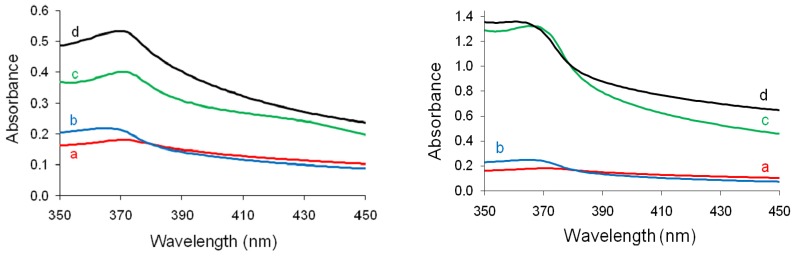
Left: UV–vis spectra of ZnO NPs obtained without PyQAs1 (**a**) and with 1% (**b**), 2.5% (**c**), and 5% (**d**) PQAs1. Right: UV–vis spectra of ZnO NPs obtained without PyQAs2 (**a**) and with 1% (**b**), 2.5% (**c**), and 5% (**d**) PyQAs2.

Figure 9 shows the UV–vis absorption spectra of saturated ethanolic solutions containing the synthesized ZnO NPs. It can be observed that in all cases absorption bands with maxima in the range of 365–370 nm were recorded, being assigned to ZnO NPs [44]. By comparing the recorded UV–vis absorption peaks one may notice that supplementary surfactant addition to the mixture of nanoparticles synthesis correlates with higher intensities of the specific absorption peaks recorded. Furthermore, the use of PyQAs2 surfactant in the synthesis of ZnO NPs leads to more intense absorption bands than for the cases where PyQAs1 surfactant was used in the synthesis process. This experimental finding may be attributed to an additional stabilization capacity induced to ZnO NPs solutions by the methoxy-containing surfactant.

**Figure 10:**
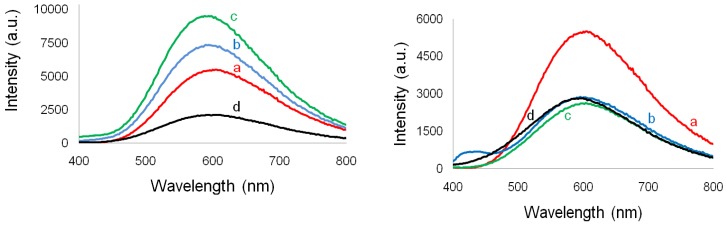
Left: Photoluminescence spectra of ZnO NPs obtained without PyQAs1 (**a**) and with 1% (**b**), 2.5% (**c**), and 5% (**d**) PyQAs1. Right: Photoluminescence spectra of ZnO NPs obtained without PyQAs2 (**a**) and with 1% (**b**), 2.5% (**c**), and 5% (**d**) PyQAs2.

The comparative effect of the two PyQAs surfactants upon the emission properties of both unmodified and PyQAs-modified ZnO NPs was also studied, and their solid-state photoluminescence spectra, collected by exciting the samples at 365 nm, are given in Figure 10. All ZnO samples were found to display wide orange-red photoemission bands, whose maxima varied in the range of 605‒594 nm in the series of ZnO NPs modified with PyQAs1, and 605‒600 nm in the series of ZnO NPs modified with PyQAs2. Despite the commonly observed UV and green emission in ZnO nanostructures [45], in our case no such phenomena were observed. The lack of UV emission is suggestive of the recombination of exciton pairs which occur rather on non-radiative recombination centers, such as dislocations, inhomogeneous impure atoms distributed within the lattice, and low-angle intergrain boundaries [46]. As far as the visible emission color is concerned, an investigation run by Ahn et al. on the origin of the visible deep centre emission(s) has suggested that the emission colour depends strongly on the synthetic and growth conditions [47]. That is why the chemical precipitation method for surface modification with organic surfactants used in the present work may have had an influence on the occurrence of orange-red emission instead of the green one. The origin of the orange-red emission peaks observed at around 600 nm is assigned to deep level defects, such as oxygen vacancies and interstitials, a phenomenon that was usually observed in the oxygen-rich samples [48]. The most intense emissions were observed in the case of ZnO NPs modified with 1 and 2.5% PyQAs, whereas all ZnO samples modified with PyQAs2 exhibited much lower photoemission intensities with respect to unmodified ZnO. Moreover, a blue-shift, more significant in the series of ZnO NPs modified with PyQAs1, running from 605 nm in ZnO-PyQAs1-5% to 594 nm in ZnO-PyQAs1-2.5%, and less significant in the series of ZnO NPs modified with PyQAs2, running from 604 nm in unmodified ZnO NPs to 600 nm in all three ZnO samples modified with PyQAs2, could also be observed. The variable intensity of the observed orange–red emissions may be due to the different number of Zn(II) available sites for bonding with the surfactants. The presence of methoxy group in the structure of PyQAs2 surfactant could lead to the formation of more bonds with the Zn(II) ions, which rapidly influences on the decrease of emission intensity with increasing surfactant concentration.

### 3.4. Investigation of Supernatant Solutions by LC-MS-ESI

After the separation of ZnO NPs all supernatant solutions were subjected to UV excitation at 365 nm, and the observed blue-green fluorescence determined us to make a preliminary investigation of these solutions by means of liquid chromatography coupled with electrospray ionization mass spectrometry (LC-MS-ESI) in order to elucidate the identity of the newly formed fluorescent species. Such an occurrence thus allowed to invoke the catalytic effect of zinc(II) ions, usually acting as a basic catalyst [49], towards the organic transformation of both nonfluorescent PyQAs species into new fluorescent organic species [50]. 

**Figure 11:**
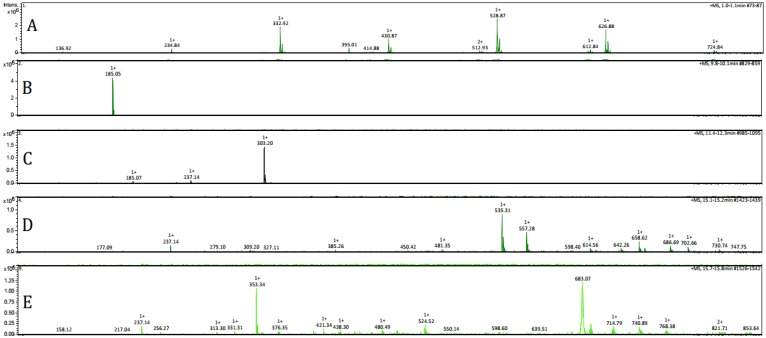
Mass spectra of the fluorescent components detected in the supernatant solutions, corresponding to the retention times peaks of 1.1 min (**A**), 9.9 min (**B**), 11.7 min (**C**), 15.1 min (**D**), and 15.7 min (**E**).

The qualitative LC-MS-ESI analysis of the products, obtained by evaporating the supernatant solutions, has shown several chromatographic peaks in all the samples. The retention times for the major compounds occurred at 1.1, 11.7, and 15.1 min when PyQAs1 was used, and at 1.1 and 15.7 min when PyQAs2 was used, thus suggesting the formation of three different fluorescent species in the ZnO-PyQAs1 series and two different fluorescent species in the ZnO-PyQAs2 series. The mass spectra profiles, recorded for the corresponding chromatographic peaks (Figure 11), allowed to suggest that the fragmentations of the newly formed fluorescent compounds take place in several unexpected cleavage sites and charge positions. For all the samples, the mass spectra shows a peak at m/z 185.05 (retention time: 9.9 min), which is characteristic to bispyridine ethane fragment (Figure 11B). Several other-charged peaks related to dimeric units were also observed in the mass spectra of the ZnO-PyQAs1 supernatant solutions’ reactions.

The overall fragmentation is notably influenced by certain structural features of the pyridinium ion [51]. Indeed, in the m/z range of 100–700, different peaks corresponding to the mass of adducts, monomeric, or dimeric units were detected (Table 5). The intensities of the signals are variables, depending on the ability of the molecule to reach the detector. The characteristic signals for adducts can be associated with heterocyclic mass fragments more (m/z 658) or less (m/z 626) aromatic. The presence of species in solution with acetate units (m/z 303) is also likely, these fragments appearing in the sample with 5% PyQAs1. Similar fragmentation behavior is observed in the mass spectra of the ZnO-PyQAs2 supernatant solutions. The ion at m/z 626 is the same as in series of ZnO-PyQAs1, but new fragments with m/z 683 and 353 appear at the retention time of 15.7 min.

**Figure 12:**
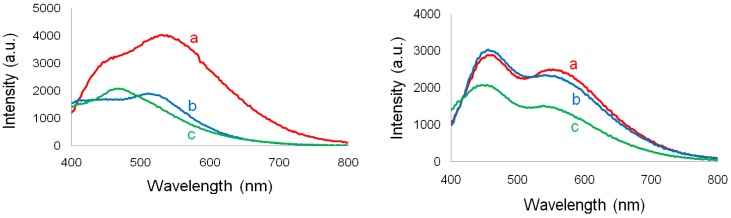
Left: Fluorescence spectra of the supernatant solutions of ZnO NPs obtained with 1% (**a**), 2.5% (**b**), and 5% (**c**) PyQAs1. Right: Fluorescence spectra of the supernatant solutions of ZnO NPs obtained with 1% (**a**), 2.5% (**b**), and 5% (**c**) PyQAs2. Excitation wavelength: 365 nm.

Figure 12 shows the fluorescence spectra of both series of supernatant solutions from which the ZnO NPs modified with PyQAs surfactants were separated. The different emissions observed in these spectra were found to be indicative of the fluorescent species that were chromatographically separated at the above-mentioned retention times. As such, the maximum emission band located at 455 nm, observed in the solution of ZnO-PyQAs1-1% and in the three solutions of ZnO-PyQAs2 series, was associated to the fluorescent component (A) (Table 4). The maximum emission band located at 530 nm, observed in the solutions of ZnO-PyQAs1-1% and ZnO-PyQAs1-2.5%, was associated to the fluorescent component (C), whereas the maximum emission band at 466 nm, observed in the solution of ZnO-PyQAs1-5%, was associated to the fluorescent component (B) (Table 4). These different results, attained in the case of ZnO-PyQAs1 series, are suggestive of the fact that, although the concentration of catalytic Zn(II) ions was the same in all three syntheses, the triggering factor for the generation of such different fluorescent species was the variable concentrations of PyQAs1 surfactant. Such an observation was no longer found valid in the case of ZnO-PyQAs2 series, since all three solutions exhibited the same emission maxima. While the maxima located at 455 nm was associated to the same fluorescent component (A) as the one detected in the first series, the maxima located at 550 nm was associated to the fluorescent component (D) that contains the fragment (E) (Table 4). In the case of this series, despite using the same concentration of catalytic Zn(II) ions and variable concentrations of PyQAs2 surfactant, the triggering factor for maintaining the same fluorescent components in all three syntheses was the presence of methoxy group. The electron-donating and electron-withdrawing substituents are shown to influence the optical properties of the compounds, which are observed in the shift of absorption maxima. Due to the presence of an electron-donating group (OCH_3_) at position 4 of the phenyl ring in PyQAs2, a slight bathochromic shift was observed in comparison with the unsubstituted PyQAs1. This bathochromic shift is caused by the increasing conjugation system due to overlapping of electrons pair of methoxy group with the π-orbitals of aromatic ring [52,53]. The increasing of an intramolecular charge transfer character of molecules would thus lead to an aggregation process of PyQAs surfactants with ZnO NPs.

## 4. Conclusions

Two cationic surfactants from the class of quaternary ammonium salts bearing the 1,2-bis(4-pyridyl)ethane core have been introduced into the surface modification practice of zinc oxide nanoparticles, and the comparative effect upon the size and photoluminescent properties was studied. Concomitantly with the formation of ZnO NPs, the catalytic transformations of the two nonfluorescent cationic surfactants into new fluorescent compounds was highlighted. The results achieved in the present work would thus pave the way towards the application of catalytic ZnO NPs in the preparation of new organic compounds with adjustable fluorescent properties. Further studies (unpublished results) are being conducted in order to isolate, through column chromatography, and characterize the content of fluorescent supernatant solutions, along with the elucidation of the catalytic mechanism and effect of ZnO NPs in the synthesis of new fluorescent heterocyclic compounds.

## Figures and Tables

**Table 1:** Comparative values of average crystallite size and FWHM values for the ZnO samples obtained by the recipe utilizing the organic agent PyQAs1.

Sample Code	Crystallite Size (nm)	FWHM (deg)
ZnO	14.666 ± 0.092	0.595 ± 0.004
ZnO-PyQAs1-1%	14.740 ± 0.097	0.592 ± 0.004
ZnO-PyQAs1-2.5%	22.966 ± 0.182	0.380 ± 0.003
ZnO-PyQAs1-5%	20.437 ± 0.122	0.427 ± 0.003

**Table 2:** Comparative values of average crystallite size and FWHM values for the ZnO samples obtained by the recipe utilizing the organic agent PyQAs2.

Sample Code	Crystallite Size (nm)	FWHM (deg)
ZnO	14.666 ± 0.092	0.595 ± 0.004
ZnO-PyQAs2-1%	15.172 ± 0.095	0.575 ± 0.004
ZnO-PyQAs2-2.5%	15.666 ± 0.089	0.557 ± 0.003
ZnO-PyQAs2-5%	13.998 ± 0.508	0.578 ± 0.005

**Table 3:** Absorption bands from the FTIR spectra of ZnO NPs and correlations with vibrations of functional groups [37,38].

Absorption Bands (cm^−1^)		Vibrations Correlated with Absorption Bands
Unmodified ZnO NPs	ZnO NPs Modified with 5% PyQAs2	
3430	3435	O–H vibrations band absorbtion
	2839, 2917, 2939, 3026, 3132	asymmetric and symmetric stretching of C–H bond
	1681	C=O stretching vibrations
	1605, 1644	C–C and C–H wag vibrations in pyridinium ring
1571	1585	C–O stretching carboxyl groups
	1521, 1512	Aromatic ring vibrations
1429	1415	C–O stretching carboxyl groups –CH_2_– bending vibrations, aromatic ring vibrations
	1348	C–H wag vibrations in pyridinium ring
	1248, 1314, 1325	pyridinium salts vibrations
	1031, 1175, 1202	C–H wag vibrations in pyridine
1022	1010	Zn–O bond vibrations, *o,o,p* C–H bend in pyridinium ring
	987	*o, o, p* C–H bend in pyridine
875	872	–CH_2_– rocking vibrations
	810, 849	C–H bond vibrations in disubstituted (para) benzene vibrations
	611	in plan ring vibrations
442	455	Zn–O bond vibrations

**Table 4:** Correlations of peaks positions in the Raman spectra of formed ZnO NPs with vibrations of functional groups [41,42].

Peak Position (cm^−1^)			Vibrations Correlated with Absorption Bands
Unmodified ZnO NPs	ZnO NPs Modified with 5% PyQAs1	ZnO NPs Modified with 5% PyQAs2	
444, 521, 582	445, 520, 583	444, 520, 583	Zn–O vibration
634, 671, 701	637, 665, 701	637, 670, 702	O–H wag vibrations of ethanol hydrogen bonded
720	721	720	C=O deformation vibration
816	809	876	CH_2_ rocking vibrations
932, 951, 976, 997, 1053, 1082, 1131	934, 953, 978, 997, 1052, 1084, 1131	935, 952, 978, 997, 1053, 1084, 1136	aromatic ring vibrations, C–C stretching vibrations for sp^3^ carbon, –CH_2_– twisting vibrations
1156	1157, 1195, 1233	1170, 1186, 1234	methyl rocking vibrations
		1435	symmetric bending vibrations of methoxy group
1477	1477	1478	CH_2_ bending vibrations
1507	1507	1507	aromatic ring vibrations, C–C stretching vibrations for sp^3^ carbon, –CH_2_– twisting vibrations
1588, 1725, 1784, 1817, 1892, 1925, 1971, 1995	1594, 1722, 1786, 1817, 1891, 1926, 1971, 1994	1591, 1723, 1785, 1819, 1892, 1925, 1971, 1995	C=O symmetric stretching vibrations (carbonyl compounds)

**Table 5:** Retention times, m/z values of the prominent ions from the mass spectra of supernatant solution, and the corresponding fragment structures of the fluorescent species.

Retention Time (min)	m/z Values	Proposed Fragment Structures	
1.1	626.88	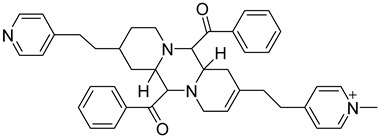	(A)
11.7	303.20	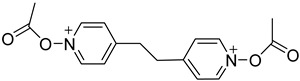	(B)
15.1	658.62	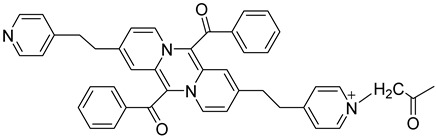	(C)
15.7	682.99	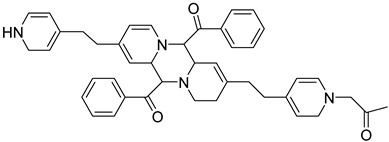	(D)
	353.34	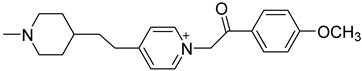	(E)
